# May Functional Imaging be Helpful for Behavioral Assessment in Children? Regions of Motor and Associative Cortico-Subcortical Circuits Can be Differentiated by Laterality and Rostrality

**DOI:** 10.3389/fnhum.2015.00314

**Published:** 2015-06-02

**Authors:** Julia M. August, Aribert Rothenberger, Juergen Baudewig, Veit Roessner, Peter Dechent

**Affiliations:** ^1^MR-Research in Neurology and Psychiatry, Georg-August-University of Goettingen, Goettingen, Germany; ^2^Department of Child and Adolescent Psychiatry, Georg-August-University of Goettingen, Goettingen, Germany; ^3^Department of Child and Adolescent Psychiatry, Carl Gustav Carus University of Dresden, Dresden, Germany; ^4^Department of Cognitive Neurology, MR-Research in Neurology and Psychiatry, Georg-August-University of Goettingen, Goettingen, Germany

**Keywords:** children, finger tapping, motor circuit, associative circuit, laterality, fMRI

## Abstract

**Background:**

Cortico-subcortical circuits are organized into the sensorimotor, associative, and limbic loop. These neuronal preconditions play an important role regarding the understanding and treatment of behavioral problems in children. Differencing evidence argues for a lateralized organization of the sensorimotor loop and a bilateral (i.e., non-lateralized) organization of the associative loop. However, a firm behavioral–neurobiological distinction of these circuits has been difficult, specifically in children.

**Objectives:**

Thus, the aim was a comprehensive functional visualization and differentiation of the sensorimotor and the associative circuit during childhood. As a new approach, laterality and rostrality features were used to distinguish between the two circuits within one single motor task.

**Methods:**

Twenty-four healthy boys performed self-paced index finger tapping with each hand separately during functional magnetic resonance imaging at 3 Tesla.

**Results:**

A contrast analysis for left against right hand movement revealed lateralized activation in typical sensorimotor regions such as primary sensorimotor cortex, caudal supplementary motor area (SMA), caudal putamen, and thalamus. A conjunction analysis confirmed bilateral involvement of known associative regions including pre-SMA, rostral SMA, and rostral putamen.

**Conclusion:**

A functional visualization of two distinct corticostriatal circuits is provided in childhood. Both the sensorimotor and associative circuit may be discriminated by their laterality characteristics already in minors. Additionally, the results support the concept of a modified functional subdivision of the SMA in a rostral (associative) and caudal (motor) part. A further development of this approach might help to nurture behavioral assessment and neurofeedback training in child mental health.

## Introduction

Connections between cortex, basal ganglia, and thalamus are organized in circuits. A model first hypothesized by Alexander et al. (1986) proposes the existence of parallel cortico-basal ganglia-thalamo-cortical loops. Within these loops, specific portions of the involved brain structures are thought to be interconnected in an anatomically and functionally segregated manner, divided according to a tripartite model: sensorimotor, associative, and limbic portions convey information between corresponding cortical and subcortical areas (Selemon and Goldman-Rakic, 1985; Alexander and Crutcher, 1990; Parent, 1990; Parent and Hazrati, 1995; Nakano et al., 2000; Postuma and Dagher, 2006; Di Martino et al., 2008). In childhood, these considerations are reflected in the evidence-based neurobiological developmental model of attention deficit/hyperactivity disorder (ADHD) by Sagvolden et al. (2005). Hence, a task-related visual differentiation of these circuits by functional imaging could be a good basis to better understand and evaluate the brain–behavior relationship in children.

In order to keep things simple, we focused experimentally on two circuits only. The sensorimotor brain circuit seems to be lateralized, while the associative circuit shows no lateralization (Kunzle, 1975; Wiesendanger et al., 1996; Gerardin et al., 2003; Lehericy et al., 2004a,b).

In order to distinguish the sensorimotor and the associative circuit in adults by *in vivo* imaging [e.g., functional magnetic resonance imaging (fMRI) and positron emission tomography (PET)], researchers varied motor task characteristics (amplitude, frequency, complexity) and thus tried to manipulate either circuit specifically (Sadato et al., 1996a,b; Boecker et al., 1998; Deiber et al., 1999; Lehericy et al., 2006). However, while some authors found cortical activations belonging to the sensorimotor and the associative circuit, respectively, no circuit-specific activation was reported for the basal ganglia (Grefkes et al., 2008).

Although much knowledge has been obtained, cortico-subcortical circuits are not yet fully understood (Kim et al., 2013; Oguri et al., 2013), specifically in childhood. Hence, in this study, we set up a comprehensive experimental design in children accounting for limitations of prior *in vivo* studies conducted mainly in human adults. We decided to focus on both cortical and basal ganglia structures, to use a motor task involving the dominant and the non-dominant hand, and to investigate *both* hemispheres with focus on laterality. Furthermore, we aimed at demonstrating both the sensorimotor and the associative circuit within a single motor task identical for each hand in order to facilitate comparison and specification^1^.

The study design and the movement task were kept simple in order to evaluate, if differentiation of the two circuits could be reached already with such a practical approach and to minimize interindividual variability. We are aware that a more sophisticated design (e.g., externally versus internally triggered movements) might have improved circuit specificity, but additional conditions were not planned and thus this must be seen as a limitation. A robust finger-tapping task for each hand separately was set up in blocked design. In order to include aspects activating associative brain structures, subjects conducted the finger tapping in a self-paced manner (Rao et al., 1996, 1997; Boecker et al., 1998; Deiber et al., 1999; Debaere et al., 2003; Witt et al., 2008), this includes motor planning (associative/cognitive aspect) and motor execution (sensorimotor aspect) in a two-step sequence. The fMRI analysis was conducted, first, by identifying brain regions which are involved in left as well as right unimanual movement, and thus being possibly part of the associative circuit. Second, it was supposed to detect areas being side-specific, i.e., areas that are more active during *either* left hand *or* right hand movement. Because of the known somatotopic organization and unilateral dominance of the sensorimotor loop, the found regions could then be considered part of it. Taking the different laterality features as the differentiating factor would be a simple and elegant method to distinguish between the two cortico-subcortical circuits, using only one motor paradigm for each hand, and providing conclusions for both hemispheres. If successful, this approach might help, in the future, to better assess and treat brain–behavior relationship in mental health of children.

## Subjects and Methods

### Subjects

We examined 26 healthy right-handed boys (mean age 11.8 years ± 1.1 years; age range 10.2–14.1 years). All were free of neurological or psychiatric disorders and none reported current or past use of psychoactive medication. Handedness was assessed by a test of laterality (Edinburgh Handedness Inventory, Oldfield, 1971); all subjects were strongly right-handed (mean score 94.9, standard deviation 14.4). Subjects had been recruited from local schools in the context of a magnetic resonance imaging (MRI) study comparing boys with Tourette Syndrome (TS) to healthy boys (Roessner et al., 2009, 2010, 2011, 2012). Informed consent was obtained from all subjects as well as from their parents. The study was approved by the local Ethics Committee.

Two of the subjects had to be excluded from the sample after data acquisition; one because of excessive head motion during the scanning session, the other one because of a tumor in the orbita which had not been known before.

### Task

We used a simple unilateral index finger-tapping paradigm in blocked design. The task was conducted with each hand separately, the order of hands being randomized across subjects. A visual display was presented via a set of magnetic resonance (MR)-suited liquid crystal display (LCD) goggles (Resonance Technology, Northridge, CA, USA) showing a red or green dot, respectively. During the red dot period, subjects were instructed to simply fixate the dot (rest condition). During the green dot period, they were instructed to tap their index onto a rigid board rhythmically (movement condition). Although subjects were instructed to perform the task at a frequency of approximately 2 Hz, the tapping was supposed to be conducted in a self-paced manner. All subjects practiced the task before scanning. Eight alternations of motor execution (12 s) and rest (18 s) were performed. With an initial rest period (18 s), the total measuring time for one experiment was 4 min 18 s.

All subjects were able to perform the finger-tapping task easily. Mirror movements or serious errors could not be observed by visual online monitoring.

The tapping was explicitly kept easy in order to involve both circuits (planning = associative; execution = sensorimotor). We therefore did not dictate a rhythm. Although performance was not measured visual inspection reflected that all the subjects were performing well with each hand and without recognizable differences. Unfortunately, we cannot differentiate quantitatively between right and left hand or calculate correlations between behavior and lateralization.

### Magnetic resonance imaging

Magnetic resonance imaging was performed at 3 Tesla (Magnetom Trio, Siemens Healthcare, Erlangen, Germany) using the standard eight channel-phased array head coil. Subjects lay supine inside the magnet bore, with foam cushions to prevent excessive head motion. Headphones were worn for noise protection as well as to allow communication between the experiments. Heart rate and blood oxygen saturation were monitored throughout.

Initially, an anatomical T1-weighted MR dataset was acquired covering the whole head at 1 mm^3^ isotropic resolution [3D Turbo FLASH, repetition time (TR): 1950 ms, inversion time: 1100 ms, echo time (TE): 3.92 ms, flip angle: 12°]. Functional imaging was performed using a T2*-sensitive gradient-echo EPI technique with an in-plane resolution of 2 × 2 mm^2^ (TR: 2000 ms, TE: 36 ms, flip angle: 70°, acquisition matrix: 96 × 128). One-hundred twenty nine volumes of 22 sections at 4 mm thickness angulated in an axial-to-coronal orientation, covering the whole brain, basal ganglia, and cerebellar structures, were acquired.

### Analysis

All the analyses were based on whole brain activation. Data analysis and visualization was performed using BrainVoyager QX (Brain Innovation, Maastricht, The Netherlands). Preprocessing of the functional data was done for each subject including 3D head motion correction, slice scan time correction, spatial smoothing (Gaussian kernel, 5 mm full width at half maximum), and linear trend removal. Subsequently, functional datasets were co-registered to the individual anatomical dataset and transformed into Talairach space (Talairach and Tournoux, 1988), as well as into functional voxels of 3 mm × 3 mm × 3 mm. A random effects analysis was conducted. Voxelwise data analysis was performed by means of the general linear model, using a deconvolution of the block design with a two-gamma function to model the hemodynamic response (Friston et al., 1998). First, main effects for each movement condition were calculated (left hand vs. rest, right hand vs. rest) in order to test for experimental validity of lateralization effects. In addition, a conjunction analysis as well as a contrast analysis was conducted. Corresponding *t*-maps were thresholded equally for all four group analyses at *p* < 0.001 (*t* > 3.77). Data were corrected for multiple comparisons using the cluster-size thresholding approach (Forman et al., 1995). This approach was chosen because firstly, it accounts best for spatial clustering of activated voxels, and secondly, the *p*-value can be kept constant for different analyses, making results comparable. For the chosen *p*-value, clusters were considered significant if their spatial extend was four functional voxels (equivalent to 108 mm^3^).

Functional group activation maps were overlaid onto the averaged T1-weighted anatomical dataset of all subjects in Talairach space (Talairach and Tournoux, 1988) and interpolated to 1 mm^3^ resolution. Clusters were defined as contiguous activations, and their spatial extend as well as local maxima were extracted. Assignment to anatomical brain regions was achieved using the Talairach Daemon (http://www.talairach.org/client.html), the local maximum coordinates being taken as the crucial point. In addition to the qualitative visualization of signal change in the figures, visual inspection of the time courses show the quantitative course of signal intensity for an activated brain region over the time, as obtained by the investigation itself. In other words, it is the same data underlying both the qualitative figures and the quantitative time courses, without further analysis.

## Results

Our analyses revealed two different patterns of brain activity during unimanual index finger tapping, one indicating movement side-specific involvement of brain areas, and the other one indicating common involvement in left as well as right unimanual movement. Structures showing subregions with both characteristic patterns are supplementary motor area (SMA) and basal ganglia.

**Figure 1:**
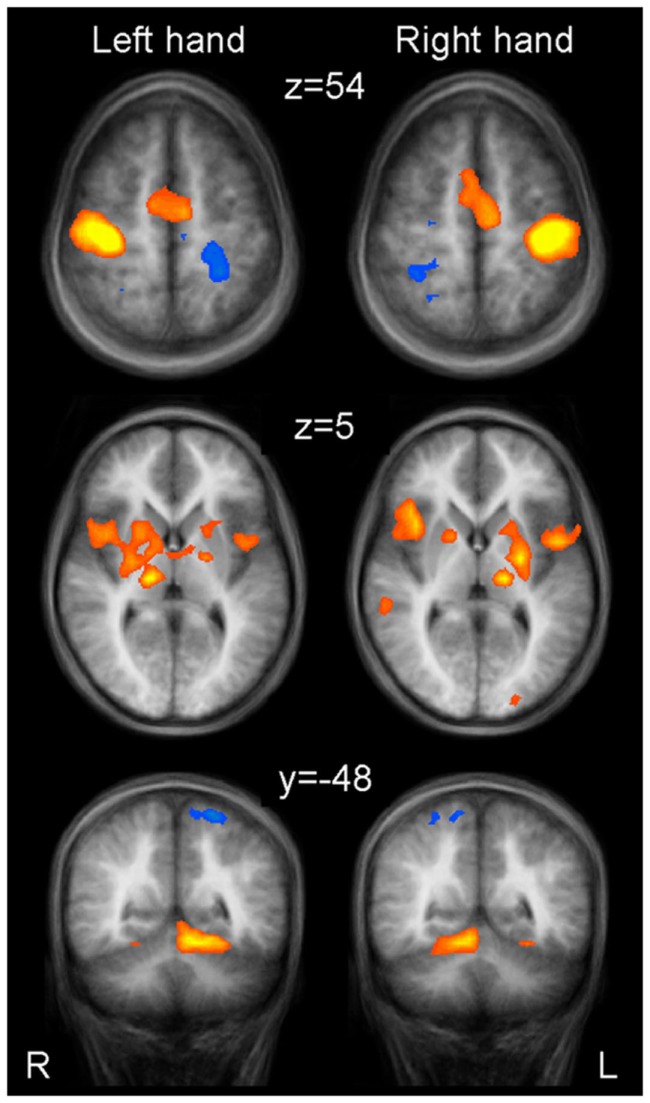
**Group activations for left hand finger tapping (left column) and right hand finger tapping (right column) versus motor rest, overlaid onto group-averaged anatomical images (*n* = 24) in Talairach space**. Significant signal changes (*p* < 0.001; corrected for multiple comparisons) are coded in yellow/orange (positive) and green/blue (negative). L, left; R, right. The Talairach coordinate in millimeter is indicated.

Activation maps were found mirror inverted for the left and right hand task, not indicating substantial differences in the patterns for the dominant and the non-dominant hand movement (Figure 1; Table 1). At cortical level, the network included activations in the primary sensorimotor cortex (SMC) contralateral to the moved index finger, bilateral SMA, pre-SMA, and anterior cingulate cortex (ACC). Further, bilateral basal ganglia structures, the contralateral thalamus as well as the ipsilateral cerebellum were activated, as well as bilateral insula and contralateral secondary somatosensory cortex (SII). Moving the index of the dominant hand produced bilateral dorsolateral prefrontal cortex (DLPFC) activation, whereas activation in left hand index movement was limited to the contralateral DLPFC. In the basal ganglia, a clear pattern was observed consisting of activation in the contralateral putamen expanding the whole rostrocaudal axis, the anterior part of the ipsilateral putamen, as well as the contralateral globus pallidus. When moving the left index, activation in the ipsilateral globus pallidus could also be detected.

**Table 1:** **Whole brain random effects group analysis**.

Region	Hemisphere	Left hand finger tapping					Right hand finger tapping				
		Talairach			Max	Size	Talairach			Max	Size
		*x*	*y*	*z*	*t* − value	(mm^3^)	*x*	*y*	*z*	*t* − value	(mm^3^)
**Main effects: brain regions activated during unilateral finger tapping Cortical motor areas**											
Pre- and postcentral gyrus	L	–	–	–	–	–	−30	−22	52	13.81	11,342
(primary sensorimotor cortex)	R	30	−25	58	14.45	9769	–	–	–	–	–
Medial frontal gyrus (SMA)	L/R	−6	−10	55	7.06	7936^a^	−6	−13	55	6.47	6947^a^
Medial frontal gyrus (pre-SMA)	L/R	0	2	46	6.4		3	5	49	6.53	
Anterior cingulate cortex	L/R	3	17	34	4.76		−3	−4	40	6.87	
Middle frontal gyrus (DLPFC)	L	–	–	–	–	–	−30	35	31	5.03	508
	R	30	41	37	5.22	586	30	41	34	6.95	2203
Postcentral gyrus	L	−24	−37	61	−6.04	4270	–	–	–	–	–
(somatosensory cortex; SI)	R	–	–	–	–	–	30	−37	46	−5.85	2729
Postcentral gyrus	L	–	–	–	–	–	−39	−22	22	5.98	1792
(somatosensory cortex; SII)	R	39	−22	25	4.86	164	–	–	–	–	–
**Insula and subcortical areas**
Insula	L	−39	2	4	5.69	6027^a^	−45	2	7	7.77	2163
Rostral putamen	L	−18	8	4	4.76		−18	5	16	6.05	8866^a^
Caudal putamen	L	–	–	–	–	–	−24	−7	7	9.31	
Globus pallidus	L	−18	−7	7	5.79		−18	−7	7	5.63	
Thalamus	L	–	–	–	–	–	−15	−19	10	8.72	
Nucleus subthalamicus	L	–	–	–	–	–	−3	−19	−8	5.13	
Insula	R	42	5	10	6.72	17,751^a^	36	14	4	8.01	4432
Rostral putamen	R	18	8	10	6.25		15	5	7	6.06	985
Caudal putamen	R	24	−10	4	6.44		–	–	–	–	–
Globus pallidus	R	18	−7	1	9.05		–	–	–	–	–
Thalamus	R	15	−19	7	9.81		–	–	–	–	–
Nucleus subthalamicus	R	9	−19	−5	4.91		–	–	–	–	–
**Cerebellum**
	L	−12	−49	−14	8.73	7818	–	–	–	–	–
	R	–	–	–	–	–	12	−46	−14	8.9	7051

*Activation differences were considered significant at *p* < 0.001, corrected for multiple comparison via cluster-size thresholding at 4 voxels (108 mm^3^). Coordinates are in millimeter according to the Talairach and Tournoux atlas. L, left; R, right; SMA, supplementary motor area; DLPFC, dorsolateral prefrontal cortex; SII, secondary somatosensory cortex*.

*^a^Summed activation of contiguous zones; the included brain regions are listed in the lines following the sum*.

Signal decreases were observed in postcentral areas ipsilateral to the moved index finger.

**Figure 2:**
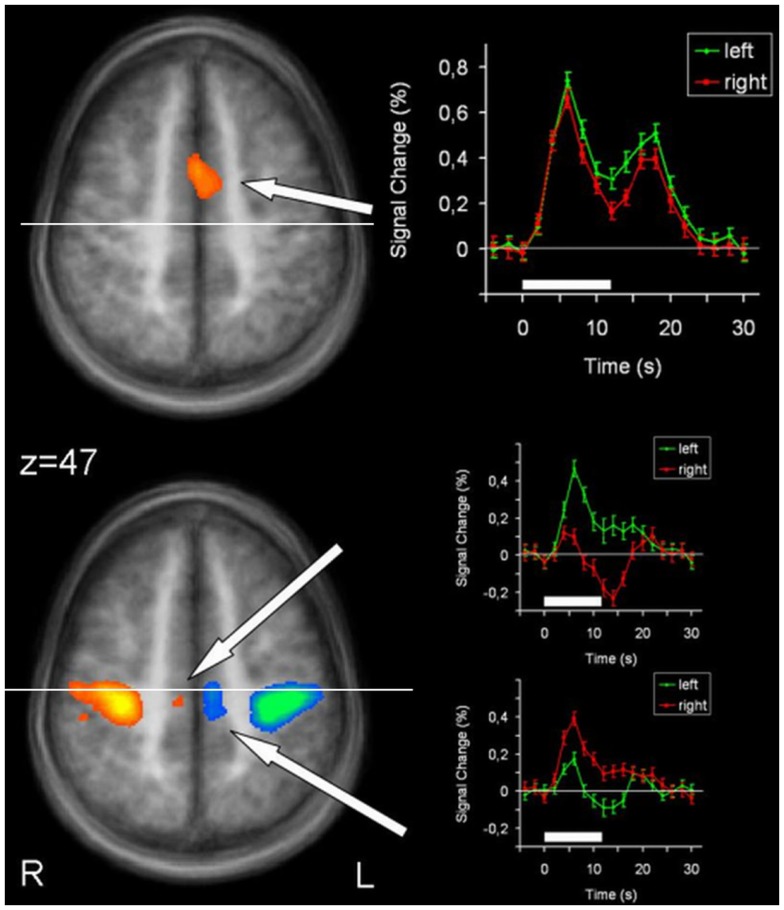
**Conjunction analysis (top) and contrast analysis (bottom) of left and right hand finger movements**. In the contrast analysis, regions more active during left hand movement are labeled in yellow/orange, those more active during right hand movement are labeled in green/blue. Time courses represent from top to bottom signal changes of rostral SMA and caudal SMA, for left hand (green) and right hand (red) finger tapping. The white bars indicate the interval of the movement condition. The white line indicates the level of the VPC-line (*y* = −22). *p* < 0.001. L, left; R, right. The Talairach coordinate in millimeter is indicated.

**Figure 3:**
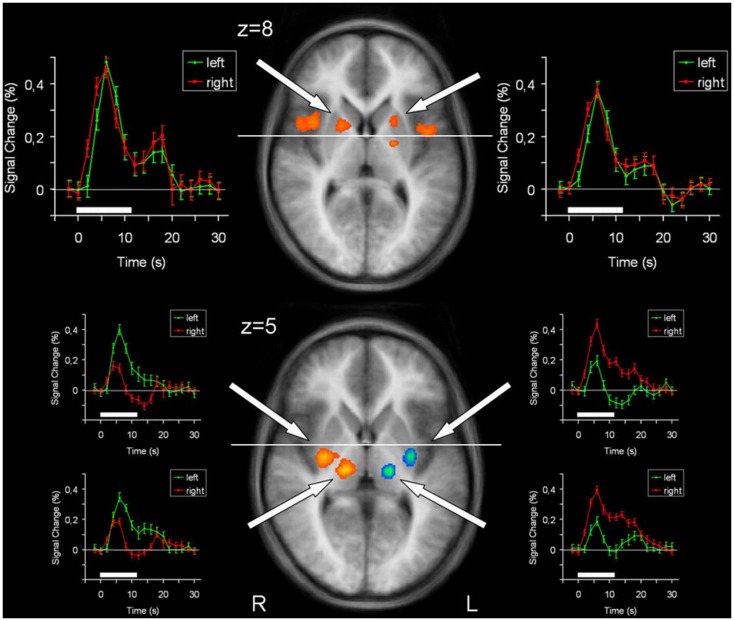
**Conjunction analysis (top) and contrast analysis (bottom) of left hand and right hand finger movements**. In the contrast analysis, regions more active during left hand movement are labeled in yellow/orange, those more active during right hand movement are labeled in green/blue. Time courses represent from top to bottom signal changes of rostral putamen, caudal putamen, and thalamus, for left hand (green) and right hand (red) finger tapping. The white bars indicate the interval of the movement condition. The white line indicates the level of the VAC-line (*y* = 0). *p* < 0.001; corrected for multiple comparisons. L, left; R, right. The Talairach coordinate in millimeter is indicated.

Brain areas activated during left hand as well as right hand finger tapping were identified via a conjunction analysis (Table 2), resulting in a nearly symmetric pattern. Activation of SMA and pre-SMA was contiguous and located in the rostral part of bilateral medial frontal gyrus (Figure 2). In the basal ganglia, the putamen showed activation bilaterally and symmetrically, but only in their rostral parts (Figure 3). Activation was also detected in the median part of the cerebellum. Further, signal increases were found in the insula bilaterally as well as in the right DLPFC. The globus pallidus was only activated in the left hemisphere. Visual inspection of the time courses obtained for the rostral SMA and the rostral putamen showed strikingly similar characteristics for contralateral and ipsilateral hand movements (Figures 2 and 3).

**Table 2:** **Whole brain random effects group analysis; *p* < 0.001; corrected for multiple comparisons via cluster-size thresholding at 4 voxels (108 mm^3^)**.

Region	Hemisphere	Talairach			Max	Size
		*x*	*y*	*z*	*t*-value	(mm^3^)
**Conjunction analysis: brain regions activated during left and right hand finger tapping**						
Medial frontal gyrus (SMAr)	L/R	−6	−13	55	6.47	4278^a^
Medial frontal gyrus (pre-SMA)	L/R	0	2	46	6.35	
Rostral putamen	L	−18	8	13	4.77	1618
	R	18	5	7	5.32	786
Globus pallidus	L	−18	−7	7	5.63	185
Cerebellum	L/R	0	−55	−14	5.03	402
Insula	L	−42	2	4	5.13	683
	R	39	8	4	5.36	1827
Middle frontal gyrus (DLPFC)	L	−30	41	25	4.08	64^b^
	R	30	41	37	5.22	464

*Coordinates are in millimeter according to the Talairach and Tournoux atlas. L, left; R, right; SMAr, rostral supplementary motor area; DLPFC, dorsolateral prefrontal cortex*.

*^a^Summed activation of contiguous zones; the included brain regions are listed in the lines following the sum*.

*^b^This activation size is below the cluster-size threshold*.

In order to detect brain regions being side-specific for finger movements of the left or right hand, respectively, we performed a contrast analysis (Table 3). A mirror inverted pattern was found. Except for the cerebellum, all effects occurred in the hemisphere contralateral to the moved hand. The SMC, caudal parts of the SMA, caudal putamen, the thalamus, and SII showed clear side specificity, as well as parts of the cerebellar hemispheres (Figures 2 and 3). Visual inspection of the time courses of caudal putamen revealed a different response behavior for contralateral and ipsilateral finger movement with signal changes for contralateral being higher than for ipsilateral movement. The timing pattern, however, was similar. Time courses for caudal putamen showed much of an analogy to thalamic time courses (Figure 3).

**Table 3:** **Whole brain random effects group analysis; *p* < 0.001; corrected for multiple comparisons via cluster-size thresholding at 4 voxels (108 mm^3^)**.

Contrast/region	Hemisphere	Talairach			Max	Size
		*x*	*y*	*z*	*t*-value	(mm^3^)

Contrast analysis: differential effects when contrasting left hand versus right hand finger tapping						
**Right hand > left hand**
Pre- and postcentral gyrus (primary sensorimotor cortex)	L	−30	−25	49	12.55	13,186
Medial frontal gyrus (SMAc)	L	−6	−22	49	5.63	952
Thalamus	L	−15	−19	4	10.16	912
Caudal putamen	L	−30	−10	4	8.99	1545^a^
Postcentral gyrus (somatosensory cortex; SII)	L	−36	−19	22	5.47	
Cerebellum	R	12	−46	−14	7.63	2047
**Left hand > right hand**
Pre- and postcentral gyrus (primary sensorimotor cortex)	R	30	−25	49	−10.21	10,819
Medial frontal gyrus (SMAc)	R	6	−25	46	−4.15	41^b^
Thalamus	R	15	−19	10	−9.1	5619^a^
Caudal putamen	R	27	−10	4	−7.13	
Postcentral gyrus (somatosensory cortex; SII)	R	39	−22	22	−7.63	
Cerebellum	L	−12	−49	−14	−7.27	2347

*Coordinates are in millimeter according to the Talairach and Tournoux atlas. L, left; R, right; SMAc, caudal supplementary motor area; SII, secondary somatosensory cortex*.

*^a^Summed activation of contiguous zones; the included brain regions are listed in the lines following the sum*.

*^b^This activation size is below the cluster-size threshold*.

## Discussion

Using a simple unilateral finger-tapping task, we were able to distinctively visualize certain regions of two functional cortico–striato–thalamo-cortical circuits in the living human brain of healthy children. Furthermore, we provide evidence for the feasibility to distinguish the sensorimotor and associative circuits by their laterality characteristics using fMRI. Additionally, our results argue for a modified functional subdivision of the SMA at this developmental stage with a rostral associative and a caudal sensorimotor compartment.

As expected, we could clearly identify the well-studied motor network involved in unilateral hand movement tasks (see also Gerardin et al., 2003; Grefkes et al., 2008; Witt et al., 2008). Main effects occurring predominantly contralaterally to the moved hand were found in the SMC and thalamus, whereas ipsilateral effects were found in the cerebellum. Although some spillover effects (e.g., via the corpus callosum) might have been taken place, the laterality effects could be clearly detected. As expected for the associative circuits, in the SMA, pre-SMA, ACC, basal ganglia, and insula, signal increases were detected bilaterally. Since both motor and cognitive activities could have been contributed to this “non-lateralization,” the finding cannot be considered as purely associative.

### Supplementary motor area

The SMA/pre-SMA/ACC region was found widely active in main effects analysis, forming contiguous activation over both hemispheres. Participation in both left and right index finger movement seems to occur in rostral parts of the SMA and pre-SMA. Side specificity can be suggested for caudal SMA. Time course analyses support this interpretation: whereas time courses for left and right hand movement were nearly identical for rostral SMA and pre-SMA, they differed for caudal SMA, with lower signal changes for ipsilateral than for contralateral hand movements.

Previous studies report similar findings (Boecker et al., 1998; Gerardin et al., 2003). This fits with the known pattern of SMA portions projecting bilaterally to the rostral striatum, whereas SMC projects mainly contralaterally to the caudal striatum.

At first glance, our activation sites in this premotor area do not fit very well with the established differentiation between SMA and pre-SMA, divided by the vertical line from the anterior commissure (VAC) (Picard and Strick, 1996, 2001; Lehericy et al., 2004a). However, extending existing concepts, our data suggest a further subdivision of the SMA itself into a rostral portion being bilaterally organized and thus possibly part of the associative circuit, and a caudal portion that would accordingly belong to the sensorimotor circuit. Evidence for such a parcelation has first been provided by Stephan et al. (1995). Studying hand/arm movements with PET, they found that caudal parts only activated during movement execution, whereas rostral parts of the SMA were also activated during its imagination. Similarly, others (Tyszka et al., 1994; Roth et al., 1996) suggested a functional differentiation, the rostral portion of the SMA (SMAr) being involved in movement imagination and the caudal portion of the SMA (SMAc) in movement execution. This model is in line with our data, especially as the border between our observed SMAr and SMAc is perfectly congruent with the vertical line from the posterior commissure (VPC) (*y* = −22).

### Putamen

Main effects analysis showed signal increases for the contralateral putamen expanding the whole rostrocaudal axis, as well as for rostral parts of the ipsilateral putamen. Conjunction analysis confirmed involvement of the bilateral rostral putamen during unilateral hand movement. Contrast analysis detected striking side specificity for the caudal putamen, suggesting distinct functional compartments of this basal ganglia structure in motor tasks. Time course analysis of the putamen was analogous to the SMA time course, with rostral parts exhibiting similar patterns for both hands and caudal parts being distinct.

Jueptner and Weiller (1998) revealed in a PET study that the learning of new motor sequences involved rostral striatum and DLPFC, i.e., associative regions, whereas pre-learned motor tasks produced activity in the caudal striatum (purely executive). Similar findings were obtained in a recent fMRI study investigating the differences in striatal activation between planning and execution of either self-generated or pre-learned movement sequences (Jankowski et al., 2009). The authors highlight that activation in the associative striatum was found bilaterally.

### Cerebellum

Likewise, cerebellar activity can be divided into bilateral and unilateral involvement. Bilateral involvement was found in the cerebellar vermis, whereas side-specific activation was located in the ipsilateral hemispheres. In previous studies, activation of the cerebellar vermis has been observed in tasks requiring associative involvement, whereas activation in the hemispheres was related to purely executive tasks (Sadato et al., 1996a; Kraft et al., 2007). Although cerebellar activity is rarely discussed in fMRI studies, Postuma and Dagher (2006) described coactivation of its portions within the framework of the proposed circuits.

Postuma and Dagher also found a functional subdivision into rostral and caudal portions for the SMA/pre-SMA and the putamen: rostral portions were coactivated and seemed to form the associative circuit together with median cerebellar parts, DLPFC, rostral insula, and dorsomedial thalamus. Caudal portions, however, were coactivated with SMC, SII, and lateral cerebellar portions, presumably composing the sensorimotor circuit. These patterns can clearly be recognized in our study. Therefore, we feel confident that our approach to identify the circuits by their laterality leads to the previously described circuits.

### General considerations

Since the study design was chosen in a way that both the sensorimotor (finger tapping) and the associative circuit (self-paced rhythm; see Boecker et al., 1998; Deiber et al., 1999; Kraft et al., 2007) be involved, activation in both sensorimotor and associative brain areas was to be expected and thus these two cortico-basal ganglio–thalamo-cortical circuits could be differentially visualized in children, a basis for further development of a neurobiologically guided exploration of behavioral dimensions.

Altered motor networks (including laterality effects) have been identified in a variety of child psychiatric disorders like ADHD (Moll et al., 2001; Roessner et al., 2004; Yordanova et al., 2013) and TS (Pourfar et al., 2011; Roessner et al., 2011, 2012). In future research on such disorders, our approach of studying the motor and associative circuits in parallel could be useful in order to find the performance level where independent or interactive neuronal activity of both circuits takes place. This could give advice for treatment development.

Comparison of our results with previous studies (mostly in adults) might be limited by our choice of subjects and the use of only one simple motor task. However, at the age of 12 years, brain size and weight are nearly 100% of their final values (Paus et al., 2001; Lenroot and Giedd, 2006; Giedd et al., 2009). Accordingly, a cross-check of our Talairach coordinates with those described by Postuma and Dagher (2006) in their meta-analysis revealed highly consistent values. Gray and white matter undergo dynamic changes until the third decade of life. However, most major tracts are significantly myelinated by early childhood. Especially basic functions such as the motor network mature earliest, both in gray and white matter (Lenroot and Giedd, 2006; Toga et al., 2006; Giedd et al., 2009). The symmetric activation maps for both, the dominant and the non-dominant hand, however, might in fact be an effect of our subjects’ age while in adults asymmetric patterns, e.g., in motor planning areas, have been described by Haaland et al. (2004).

One could also question if it is appropriate to deduce a generalized motor circuit model from a finger movement paradigm. It is true that previous studies have shown differential local involvement of brain structures depending on the underlying somatotopy. However, somatotopy seems to occur *within* the proposed portions: corticostriatal circuits can likewise be recognized in foot movement tasks (Lehericy et al., 1998; Gerardin et al., 2003).

## Conclusion

We draw three main conclusions. First, we were able to visualize differential regions of two functionally cortico–striato–thalamo-cortical circuits based on their laterality characteristics by fMRI using a simple and robust motor paradigm. The lateralized sensorimotor circuit involves SMC, SMAc, caudal putamen, thalamus, SII, and cerebellum; its projections run within the hemisphere contralateral to the moved hand – except for cerebellar connections. The associative circuit is bilaterally (i.e., non-lateralized) organized, including SMAr, pre-SMA, rostral putamen, insula, and cerebellar vermis. Second, our experimental paradigm of self-paced index finger movements of the left and right hand separately represents a new approach for the differentiation of the two circuits, potentially interesting for further research on movement problems in child psychiatric disorders and dimensions of other behavior; i.e., this approach is still experimental and far away from a “diagnostic tool.” Third, as a new finding in children, we suggest a modified concept for the functional subdivision of the SMA, the rostral part being organized bilaterally and thus possibly belonging to the associative circuit, and the caudal part showing lateralization, pointing toward its assignment to the sensorimotor circuit. This fits with similar preliminary findings in adults (e.g., Stephan et al., 1995) which have to be confirmed. A further development of this approach might help to nurture behavioral assessment and neurofeedback training in child psychiatric problems.

## Conflict of Interest Statement

Julia M. August, Jürgen Baudewig, and Peter Dechent do not have any conflicts of interest. Veit Roessner is member of an advisory board of Shire and got travel support from Novartis. He got research support from the EU, the German Research Society, the German Federal Ministry of Education and Research, the Else-Kröner-Fresenius-Stiftung, the Friede-Springer-Stiftung, the Roland-Ernst-Stiftung, the Sächsisches Staatsministerium für Soziales und Verbraucherschutz, the German Tourette-Syndrome-Association. Aribert Rothenberger is member of an advisory board and speakers’ bureau of Lilly, Shire, Medice, and Novartis. He got research and travel support and an educational grant from Shire and research support from the German Research Society.

## Footnotes

^1^The sensorimotor loop is known to include the SMC, SMA, posterior putamen, thalamus, and parts of the insula and of the cerebellum (Postuma and Dagher, 2006; Di Martino et al., 2008). These brain areas are involved in strictly executive motor control and have been shown to activate predominantly unilaterally, namely – except for the cerebellum – contralaterally to the moved hand. The sensorimotor loop is known to be organized somatotopically. Activated brain regions should not overlap for left and right unilateral hand movements. The associative loop includes regions such as pre-SMA, anterior putamen, DLPFC, and again parts of the insula and the cerebellum (Postuma and Dagher, 2006; Di Martino et al., 2008). These brain areas are involved in planning and timing of movements. In contrast to the sensorimotor loop, the associative loop is known to be *not* somatotopically organized, and activation in these regions has been reported to occur bilaterally. Hence, regions belonging to this circuit should be active during left as well as right unimanual movement, resulting in a common activation zone. Taking these pieces of information together, it should be possible to distinguish the circuits by their laterality characteristics.

## Abbreviations

ACC, anterior cingulate cortex; ADHD, attention deficit/hyperactivity disorder; DLPFC, dorsolateral prefrontal cortex; fMRI, functional magnetic resonance imaging; LCD, liquid crystal display; MR, magnetic resonance; MRI, magnetic resonance imaging; PET, positron emission tomography; SII, secondary somatosensory cortex; SMA, supplementary motor area; SMAc, caudal portion of the SMA; SMAr, rostral portion of the SMA; SMC, primary sensorimotor cortex; TE, echo time; TR, repetition time; TS, Tourette syndrome; VAC, vertical line from the anterior commissure; VPC, vertical line from the posterior commissure.
